# Transcriptome Assembly and Comparative Analysis of the Superoxide Dismutase (SOD) Gene Family in Three *Hyotissa* Species

**DOI:** 10.3390/biology15010004

**Published:** 2025-12-19

**Authors:** Xiangjie Kong, Sheng Liu, Shan Zhang, Youli Liu, Zhihua Lin, Qinggang Xue

**Affiliations:** 1 Ninghai Institute of Mariculture Breeding and Seed Industry, Zhejiang Wanli University, Ningbo 315604, China; kongxiangjie0724@163.com (X.K.); zhangshan202508@163.com (S.Z.); liuyouli@zwu.edu.cn (Y.L.); zhihua9988@126.com (Z.L.); 2 Zhejiang Key Laboratory of Aquatic Germplasm Resource, Zhejiang Wanli University, Ningbo 315100, China

**Keywords:** *Hyotissa*, de novo transcriptome, superoxide dismutase (SOD), Dominin, copper-only SOD repeat proteins (CSRPs), gene family evolution

## Abstract

The genus *Hyotissa* (family Gryphaeidae) comprises ecologically and economically important marine bivalves, yet their molecular biology remains poorly characterized. This study presents de novo transcriptome sequencing of three *Hyotissa* species—*H. sinensis*, *H. inaequivalvis*, and *Hyotissa* sp.—to systematically identify and characterize the superoxide dismutase (SOD) gene family, a crucial component of the antioxidant defense system. We identified 46 SOD genes, including both Cu/Zn-SOD and Fe/Mn-SOD types, which exhibited considerable variation in molecular properties, domain architecture, and potential phosphorylation sites. Phylogenetic analysis revealed both evolutionary conservation and diversification of SODs across species. Notably, we identified homologs of two specialized SOD types: Dominin, which showed mutations in metal-binding sites suggestive of functional divergence, and copper-only SOD repeat proteins (CSRPs), which retained copper-binding residues but lost zinc-binding capacity. These findings suggest that the SOD family in *Hyotissa* has undergone significant functional diversification, potentially as an adaptive response to their high-oxygen, high-ultraviolet reef habitats. This study provides foundational transcriptomic resources for *Hyotissa* and offers new insights into the evolution and environmental adaptation of SOD genes in marine bivalves.

## Simple Summary

The marine bivalves of the genus *Hyotissa* (family Gryphaeidae) are important for ocean ecosystems and fisheries; however, their molecular biology information is notably scarce. Our study aimed to specifically explore crucial defense genes called superoxide dismutases (SODs), which act as the body’s natural defenders against cellular damage. We discovered 46 diverse SOD genes in three *Hyotissa* species. Our analysis revealed that these genes possess both ancient, conserved features and newer, diversified characteristics. Intriguingly, we found specialized SODs like Dominin and copper-only SOD repeat proteins (CSRPs), exhibiting structural changes hinting at new roles. These variations suggest *Hyotissa*’s protective genes have adapted to perform various tasks, potentially enabling them to thrive in demanding reef environments characterized by high ultraviolet radiation and high oxygen levels. This study provides foundational transcriptomic resources for *Hyotissa* and offers new insights into the evolution and environmental adaptation of SOD genes in marine bivalves.

## 1. Introduction

The superfamily Ostreoidea, which includes the families Gryphaeidae and Ostreidae, represents a major clade of marine bivalves [1]. While Ostreidae has been relatively well-studied, Gryphaeidae diverged earlier, during the Permian–Triassic period [2]. Within Gryphaeidae, the genus *Hyotissa* has notably rich species [3], making it a valuable group for understanding evolutionary trajectories and adaptive diversification in Ostreoidea. *Hyotissa* species are widely distributed in tropical and subtropical waters of the Western Atlantic, Indo-Pacific, and Eastern Pacific [4,5]. They are typically large, with thick shells, prominent radial ribs, and unique vesicular microstructures [6,7]. The complex three-dimensional structure of their shells provides habitat for diverse microfauna, underscoring their ecological importance [8]. Additionally, the adductor muscle of *Hyotissa* species—comprising over half of their soft tissue—is highly valued for its quality, giving these species significant economic value and aquaculture potential [9].

Superoxide dismutase (SOD) is a key antioxidant enzyme that catalyzes the dismutation of superoxide anion (O_2_^−^) into oxygen and hydrogen peroxide (H_2_O_2_), thereby mitigating reactive oxygen species (ROS) and reducing oxidative damage [10,11,12]. SODs are classified based on their metal cofactors into Cu/Zn-SOD, Fe/Mn-SOD, and Ni-SOD types [13]. In eukaryotes, Cu/Zn-SODs are further divided into intracellular (icCu/Zn-SOD) and extracellular (ecCu/Zn-SOD) forms, which differ in subcellular localization and function [14]. Recent studies have revealed considerable expansion and functional diversification of the SOD gene family in some invertebrates [15,16,17]. For instance, the extracellular Cu/Zn-SOD homolog Dominin in oysters appears to have lost canonical SOD activity, acquiring new roles in metal binding and immune regulation [18,19,20]. Similarly, the recently discovered copper-only SOD (Cu-only SOD) and its multi-domain form, copper-only SOD repeat proteins (CSRPs), retain conserved copper-binding sites for catalysis but have mutated zinc-binding sites, further enriching the functional diversity of the SOD family [21,22].

Unlike Ostreidae species such as the Pacific oyster (*Crassostrea gigas*), which inhabit estuarine, intertidal, or shallow marine environments with muddy or sandy substrates, *Hyotissa* species typically attach to coral reefs or other hard substrates [23,24]. These habitats are characterized by clear water, stable high salinity, warm temperatures, and intense ultraviolet radiation, leading to elevated oxidative stress [23]. Such conditions, particularly high UV exposure and temperature, promote persistent ROS production [25]. As SODs constitute the first line of defense in the antioxidant system, the pronounced oxidative stress in *Hyotissa* habitats may have driven adaptive evolution in their SOD gene family, resulting in a more robust antioxidant defense system.

Advances in high-throughput sequencing have revolutionized biological research, enabling large-scale genomics and transcriptomics projects [26]. However, despite the recent completion of the first chromosome-level genome for *H. hyotis* [27], genomic and transcriptomic data for *Hyotissa* remain scarce in public databases like NCBI. This data gap has led to species misidentification (e.g., *H. hyotis* being mislabeled as *H. sinensis*) and has hindered related research. To address this, we performed de novo transcriptome sequencing of three *Hyotissa* species and systematically identified and characterized their SOD genes. Our analysis of molecular characteristics, conservation, and specificity provides a foundation for understanding environmental adaptation and phenotypic evolution in Gryphaeidae oysters.

## 2. Materials and Methods

### 2.1. Sample Collection

**Figure 1:**
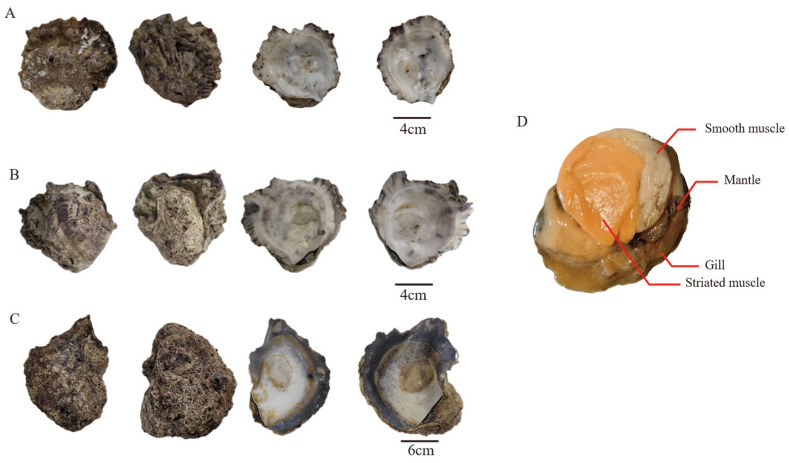
Shell morphology of three *Hyotissa* species. (**A**) *Hyotissa* sp., (**B**) *H. inaequivalvis*, (**C**) *H. sinensis*, (**D**) Schematic of tissues for transcriptomic analysis.

Five wild *Hyotissa* individuals were collected from Weizhou Island, Beihai City, Guangxi Zhuang Autonomous Region, China (21°02′4″ N, 109°06′6″ E). Based on mitochondrial 16S rRNA gene fragment sequencing, three species were identified: individuals 1 and 3 as *Hyotissa inaequivalvis*, individual 2 as *Hyotissa* sp. and individuals 4 and 5 as *Hyotissa sinensis* [28]. After collection, each oyster was dissected to separate gill, mantle, smooth muscle, and striated muscle tissues (Figure 1).

### 2.2. Total RNA Extraction and Quality Control

Total RNA was extracted from four tissues using the TRNzol Universal Reagent Kit (TIANGEN, Beijing, China) according to the manufacturer’s instructions. RNA integrity (RIN) and quality were assessed using an Agilent 5400 Bioanalyzer (Agilent, Santa Clara, CA, USA). Only RNA samples with a concentration ≥ 50 ng/µL, a RIN value > 6.0, and no detectable protein or organic solvent contamination were used for cDNA library construction.

### 2.3. Library Construction and Sequencing

Libraries were constructed and sequenced by Novogene Corporation (Beijing, China). The Fast RNA-seq Lib Prep Kit V2 (Abclonal, Wuhan, China) was used for mRNA enrichment, fragmentation, and cDNA library construction. Libraries were preliminarily quantified with a Qubit 2.0 Fluorometer (Thermo, Waltham, MA, USA), diluted to 1.5 ng/μL, and insert size was assessed with an Agilent 5400 Bioanalyzer. After confirming expected insert size, the effective concentration was quantified via qRT-PCR to ensure ≥1.5 nM. Qualified libraries were sequenced on the Illumina NovaSeq X Plus platform (Illumina, San Diego, CA, USA), generating 150 bp paired-end reads, with a target data output of 6 G for each library.

### 2.4. RNA-Seq Data Processing and Quality Control

Raw read quality was assessed with FastQC (v0.12.1) for base quality, GC content, duplication rate, overrepresented k-mers, and quality score distribution [29]. MultiQC (v1.30) aggregated quality metrics across samples [30]. Raw reads were processed with fastp (v0.19.7) [31] to remove adapter-containing reads, poly-N sequences, and low-quality reads, yielding clean reads. Q20 scores, Q30 scores, and GC content of the clean data were calculated.

### 2.5. Transcriptome Assembly

In this study, we performed independent de novo transcriptome assembly and annotation for five *Hyotissa* individuals. For each oyster, high-quality sequencing data were assembled de novo using Trinity (v2.13.2), yielding five separate transcriptomes [32]. The assembly was performed using default parameters optimized for transcriptome data. The completeness of the transcriptome assembly was assessed using Benchmarking Universal Single-Copy Orthologs (BUSCO) software (v5.5.0) with default parameters. Each assembly was compared with the mollusca_odb10 database (http://busco.ezlab.org/) (accessed on 10 December 2025) to evaluate its completeness [33]. Then, TransDecoder (v5.5.0) was used to predict the open reading frames (ORFs) of the filtered nucleotide sequences and translate them into protein sequences [34]. The longest protein sequence from the resulting protein sequence file was extracted as the input file for subsequent analysis.

### 2.6. Transcriptome Assembly and SOD Gene Identification

SOD genes were identified from transcriptomic data of five *Hyotissa* individuals. Gene IDs were labeled with individual-specific prefixes (“Hsi1-2”, “Hin1-2”, “Hsp1”) to denote species origin. Candidate SOD sequences were obtained using BLAST (v2.12.0) and HMMER (v3.2.1) with significant hits (E-value ≤ 1.00 × 10^−10^) and SOD domain matches (PF00080, PF00081, PF02777; E-value ≤ 1.00 × 10^−10^). Domain presence was verified with SMART (https://smart.embl.de/) (accessed on 15 May 2025). Genes with partial coding sequences were excluded from further analysis.

### 2.7. Molecular Characterization of SOD Family Members

Amino acid length, molecular weight (kDa), isoelectric point (pI), instability index, aliphatic index, and grand average of hydropathicity (GRAVY) were calculated using Expasy ProtParam (https://web.expasy.org/protparam/) (accessed on 15 May 2025) with default parameters [35]. Subcellular localization was predicted with WoLFPSORT (https://wolfpsort.hgc.jp/) (accessed on 15 May 2025) with default parameters [36]. Phosphorylation sites were predicted using NetPhos (v3.1) (https://services.healthtech.dtu.dk/services/NetPhos-3.1/) (accessed on 15 May 2025) [37]. The prediction score ranges from 0.000 to 1.000, and sites with a score above the threshold of 0.500 were considered positive predictions. Signal peptides and transmembrane domains were analyzed using SignalP 6.0 with default settings (https://services.healthtech.dtu.dk/services/SignalP-6.0/) (accessed on 15 May 2025) [38]. Conserved motifs were identified with MEME (https://meme-suite.org/meme/tools/meme) (accessed on 15 May 2025), with the maximum number set to 10 and other parameters as default [39].

### 2.8. Sequence Alignment and Phylogenetic Analysis

The SOD genes identified from the transcriptome data of *Hyotissa* were subjected to multiple sequence alignment with the SOD protein sequences retrieved from the NCBI database for Pacific oyster (*Crassostrea gigas*), human (*Homo sapiens*), mouse (*Mus musculus*), zebrafish (*Danio rerio*), fruit fly (*Drosophila melanogaster*), and nematode (*Caenorhabditis elegans*). The alignment was performed using MAFFT software (v7.526) with the BLOSUM62 amino acid substitution matrix [40]. The best substitution model (WAG+G) was selected using MEGA 11, and a phylogenetic tree was constructed using the Maximum Likelihood (ML) method in MEGA 11, with branch support assessed by the bootstrap method with 1000 replicates.

To further analyze the evolutionary relationships of the two specialized SOD types in oysters, Dominin and CSRP, the Dominin3 (XP_011439797.1) and CSRP (XP_034330263.1) sequences from *C. gigas* were used as a reference to search for homologous sequences in transcriptomic data from five individuals of the *Hyotissa* genus, with an E-value threshold of less than 1 × 10^−10^ and a query coverage greater than 40% as screening criteria. The homologous Dominin sequences identified from the Hyotissa genus were subjected to multiple amino acid sequence alignment with the intracellular Cg-Cu/Zn-SOD (XP_034334952.2) from *C. gigas* [20], Cg-Dominin3, human extracellular SOD (HuEcSOD, P08294), and human cytoplasmic SOD (HuCySOD, P00441) to analyze their key active sites, and a phylogenetic tree was constructed. Additionally, the individual SOD domains of *Hyotissa* CSRP were aligned with HuEcSOD (P08294), HuCySOD (P00441), and *C. gigas* intracellular Cu/Zn-SOD (XP_034334952.2) using multiple sequence alignment to analyze the conserved amino acid residues essential for copper ion coordination in their active sites.

## 3. Results

### 3.1. Technical Validation of Transcriptome Sequencing

Illumina sequencing generated 464,824,286 paired-end reads (Table 1). The number of raw reads per sample ranged from 21,172,853 to 24,393,919. MultiQC reports confirmed high base quality (Phred score > 35) and high mean quality scores across sequences. After processing with fastp, 456,109,722 high-quality clean read pairs were obtained. This corresponded to a per-sample clean read count range of 20,776,969 to 25,021,203 reads. Q20 and Q30 scores were ≥98.07% and ≥94.23%, respectively, and GC contents were within expected ranges (Table 1). After assembly, the number of transcripts across the five samples ranges from 327,624 to 379,502, with assembly continuity, as measured by N50, varying between 1324 and 1595 bp (Table S1). Additionally, BUSCO analysis revealed high completeness (Table S2). These findings show that a high-quality RNA-seq dataset was successfully generated. Raw transcriptome data are available in the NCBI SRA under accession number PRJNA1356962.

### 3.2. Identification of the SOD Gene Family

**Figure 2:**
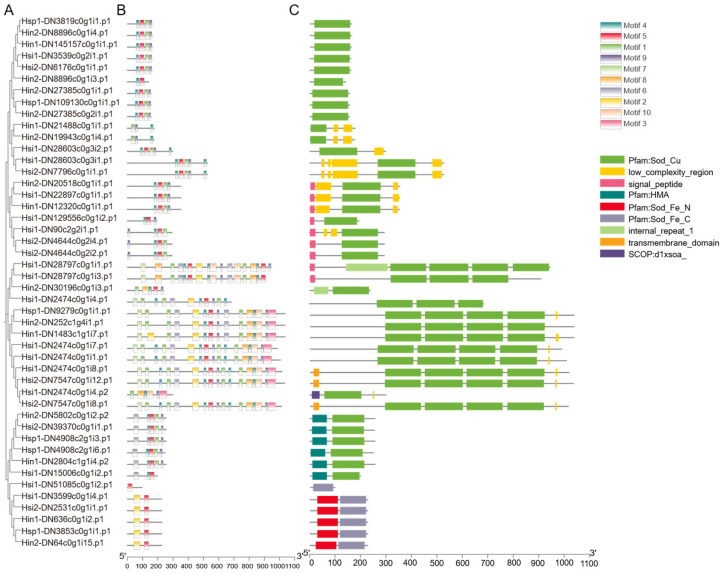
Phylogenetic, motif, and domain analysis of *Hyotissa* SODs. (**A**) ML phylogenetic tree of SOD proteins. (**B**) Conserved motifs (colored boxes). (**C**) Conserved domains. Hsi: *Hyotissa sinensis*, Hin: *Hyotissa inaequivalvis*, Hsp: *Hyotissa* sp.

A total of 46 SOD family genes were identified from the five *Hyotissa* transcriptomes: six each from Hin1 and Hsp1, ten from Hin2, sixteen from Hsi1, and eight from Hsi2 (Table 2 and Table S3). Domain analysis classified five sequences as Fe/Mn-SODs, based on the presence of N-terminal Fe/Mn-SOD α-hairpin (PF00081) and C-terminal Fe/Mn-SOD (PF02777) domains; one sequence contained only the C-terminal domain. The remaining sequences contained one to four Sod_Cu domains (PF00080) and were identified as Cu/Zn-SOD homologs. Six of these also contained a heavy metal-associated (HMA) domain (PF00403) (Figure 2).

Physicochemical properties varied widely: amino acid lengths ranged from 95 to 1035 residues, molecular weights from 11.06 to 112.32 kDa, and pI from 5.10 to 9.75 (mostly 5.5–7.8). Instability indices ranged from 14.29 to 54.06, with many exceeding 40, indicating potential instability. Aliphatic indices were generally high (>70), suggesting thermostability. All proteins had negative GRAVY values, indicating hydrophilicity. Twelve proteins possessed signal peptides, suggesting extracellular secretion or organelle localization. Phosphorylation sites ranged from 5 to 130, with Hin1-DN1483c1g1i7.p1 having the highest number, indicating potential for functional diversification.

MEME analysis identified ten conserved motifs (Figure 2B). Motif 1, associated with the Sod_Cu domain, was present in all Cu/Zn-SODs. Except for Hsi1-DN51085c0g1i2.p1, all Fe/Mn-SODs contained Motif 2 (N-terminal Fe/Mn-SOD α-hairpin) and Motif 5 (C-terminal Fe/Mn-SOD domain).

### 3.3. Phylogenetic Analysis of Hyotissa SOD

**Figure 3:**
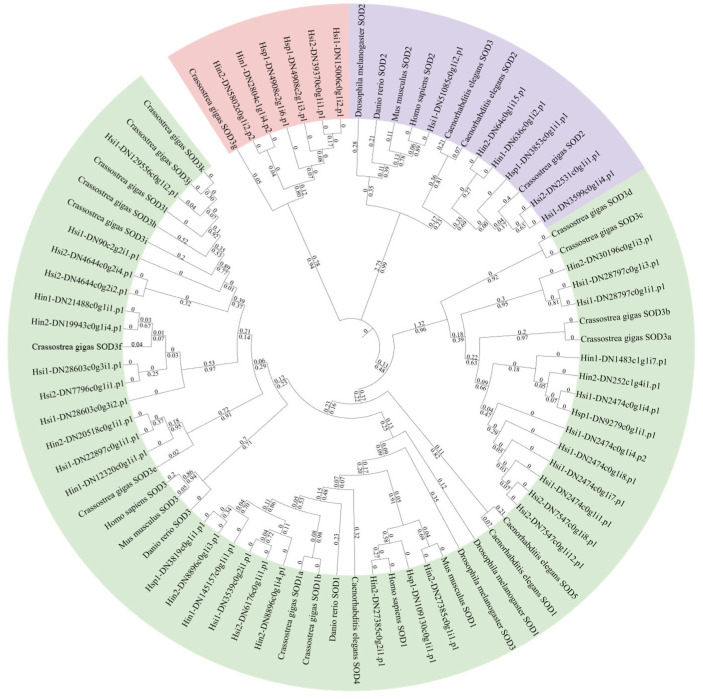
ML phylogenetic tree of SODs from *Hyotissa* and six reference species, with bootstrap values from 1000 replicates and branch lengths displayed. Green: Cu/Zn-SOD, Purple: Mn-SOD, Red: Copper chaperone for SOD. Hsi: *Hyotissa sinensis*, Hin: *Hyotissa inaequivalvis*, Hsp: *Hyotissa* sp.

A phylogenetic tree was constructed using SOD sequences from *Hyotissa* and six other species. Due to significant expansion in *C. gigas*, its SODs were designated SOD1a-b, SOD2, and SOD3a-l based on relationships with intracellular Cu/Zn-SOD (SOD1), Mn-SOD (SOD2), and extracellular Cu/Zn-SOD (SOD3). The tree revealed three major clades: Cu/Zn-SOD (48% bootstrap), Mn-SOD (99% bootstrap), and copper chaperone for SOD (94% bootstrap) (Figure 3).

### 3.4. Sequence Analysis of the ecSOD Homolog Dominin in Hyotissa

**Figure 4:**
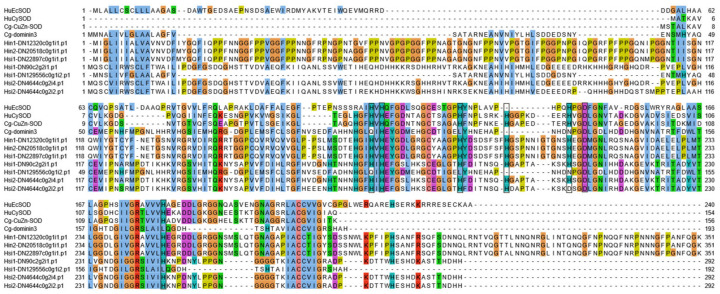
Multiple alignment of *Hyotissa* Dominin homologs with reference SODs-Cg-icCu/Zn-SOD, Cg-Dominin3, HuCySOD, and HuEcSOD. Black boxes: Metal-binding sites. Hsi: *Hyotissa sinensis*, Hin: *Hyotissa inaequivalvis*.

**Figure 5:**
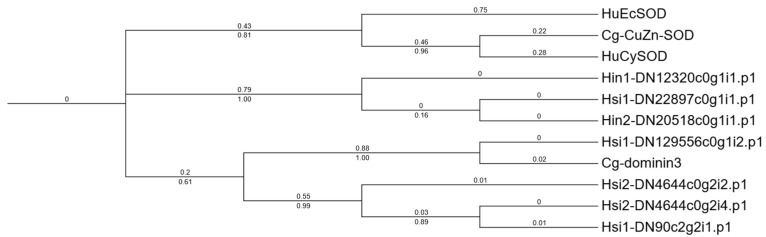
ML phylogenetic tree of *Hyotissa* Dominin homologs and reference SODs. Cg-Cu/Zn-SOD, Cg-Dominin3, HuCySOD, and HuEcSOD, with bootstrap values from 1000 replicates and branch lengths displayed. Hsi: *Hyotissa sinensis*, Hin: *Hyotissa inaequivalvis*.

Seven Dominin homologs were identified in the *Hyotissa* transcriptomes. Sequence alignment with Cg-Cu/Zn-SOD, Cg-Dominin3, HuEcSOD, and HuCySOD showed that Hsi1-DN129556c0g1i2.p1 shared five metal-binding site mutations with Cg-Dominin3, while Hsi2-DN4644c0g2i2.p1 had only one mutation. The other five homologs were identical to Cg-Cu/Zn-SOD at all seven metal-binding sites (Figure 4). Phylogenetic analysis indicated divergent evolution: one group was orthologous to *C. gigas* Dominin3, while the other formed a distinct clade (Figure 5).

### 3.5. Sequence and Domain Comparison of Hyotissa CSRPs

**Figure 6:**
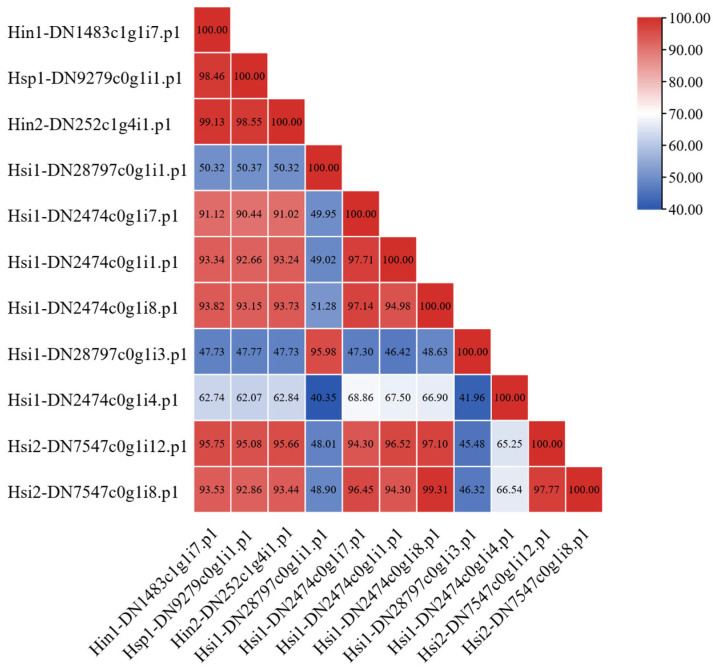
Amino acid sequence similarity of *Hyotissa* CSRPs (percent similarity shown). Hsi: *Hyotissa sinensis*, Hin: *Hyotissa inaequivalvis*, Hsp: *Hyotissa* sp.

**Figure 7:**
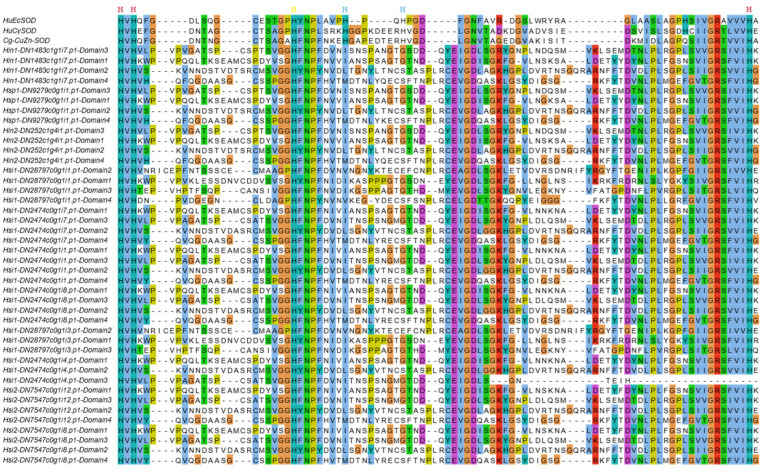
Alignment of *Hyotissa* CSRP Cu-SOD domains with reference SODs. Red: Cu-binding sites, Yellow: Dynamic histidine, Blue: Zn-binding sites. Hsi: *Hyotissa sinensis*, Hin: *Hyotissa inaequivalvis*, Hsp: *Hyotissa* sp.

Eleven CSRPs were identified among the 46 SOD genes. Pairwise amino acid identity ranged from 40.35% to 99.31% (Figure 6). The amino acid coordinates for all Cu-SOD domains are listed; two CSRPs were found to contain only three such domains, while the remaining nine contained the typical four-domain architecture (Table S4). Except for two sequences with three Cu-SOD domains, all others had four. Alignment showed that *Hyotissa* CSRP domains retained the five conserved residues for Cu^2+^ coordination but had mutations in two histidine residues critical for Zn^2+^ binding (Figure 7).

## 4. Discussion

Despite extensive omics data for Ostreidae (e.g., *C. gigas*, *C. hongkongensis*) [24,41], *Hyotissa* (Gryphaeidae) remains underrepresented in public databases—with only *H. hyotis*’ genome recently published [27]. This scarcity has hindered studies of trait-associated genes (e.g., stress resistance, biomineralization) and molecular mechanisms of adaptation, and our de novo transcriptomes of three *Hyotissa* species address this gap, providing a resource for functional gene mining and evolutionary research.

The 46 identified SOD genes (Cu/Zn-SOD, Fe/Mn-SOD) exhibit broad variation in domains, physicochemical properties, and phosphorylation sites—suggesting functional flexibility. Phylogenetic alignment with model organisms confirms their evolutionary conservation while highlighting Hyotissa-specific diversification, likely driven by coral reef stressors (high UV, oxidative stress). Since such stress results in elevated ROS levels, antioxidant enzymes are upregulated in response to stress stimuli in order to protect the organism from cellular damage [42,43]. The Hyotissa-specific SOD lineages may facilitate a more robust and tiered defense system, allowing these bivalves to thrive in highly fluctuating reef habitats.

Dominin, a major plasma protein in *Crassostrea virginica*, constitutes over 40% of plasma and pallial cavity fluid protein, indicating a critical physiological role. Despite having a Cu/Zn-SOD domain, mutations in five metal-coordinating histidines abolish SOD activity [18,19,20]. Among the seven Dominin homologs identified in *Hyotissa*, phylogenetic analysis revealed two groups: one orthologous to *C. gigas* Dominin3 and another distinct clade. Beyond descriptive sequence alignment—which showed varying mutations in metal-binding sites—we infer potential functional divergences. Sequence alignment showed varying metal-binding site mutations: Hsi1-DN129556c0g1i2.p1 shared five mutations with Cg-Dominin3, likely losing SOD activity, while Hsi2-DN4644c0g2i2.p1 had only one mutation, potentially preserving SOD activity [20]. The other five homologs contain fully conserved metal-binding sites, indicating they may function as active extracellular SODs (EC-SODs). This contrasts sharply with Ostreidae oysters, which appear to have evolved Dominin primarily as a non-enzymatic protein, possibly leaving them without an EC-SOD-active form. These differences suggest divergent evolutionary paths in immune-related protein function between oyster families, which may reflect adaptation to distinct physiological or environmental challenges.

CSRPs, multi-domain copper-only antioxidant enzymes, have been identified in various oysters [17]. They possess SOD activity and are upregulated under stress, indicating a role in extracellular antioxidant defense [22]. We identified several CSRP homologs, four with Cu-SOD domains and two with three domains. The latter deviates from the typical four-domain architecture in Ostreidae, possibly due to incomplete assembly. One domain in Hsi1-DN2474c0g1i4.p1 lacked a full set of conserved residues. These are tentatively classified as “candidate CSRP genes with three Cu-SOD domains,” requiring full-length cDNA validation. If confirmed, a three-domain CSRP in *Hyotissa* would suggest that the four-domain structure is not essential for antioxidant defense under certain evolutionary pressures. From an evolutionary perspective, the reduction in repeated domains often correlates with functional specialization, structural stability adjustment, and adaptive optimization [44]. The three-domain architecture in *Hyotissa* may therefore not represent a simple loss but a refined adaptation to its unique habitat. This streamlined form could alter protein–protein interactions or ROS-scavenging kinetics, representing an evolutionary trade-off that balances antioxidant capacity with the specific environmental pressures characteristic of its niche, such as thermal fluctuations, hypoxia, or osmotic stress.

Finally, this study has limitations that should be addressed in future research: the small sample size (*n* = 5 individuals) may limit the representativeness and generalizability of the findings, the three-domain CSRP structure requires full-length cDNA cloning to rule out assembly gaps, SOD activity assays (e.g., for Dominin and CSRPs) are needed to confirm enzymatic capacity, and integrating additional *Hyotissa* genomes will clarify SOD family evolution across Gryphaeidae.

## 5. Conclusions

In summary, this study provides high-quality transcriptomic resources for three *Hyotissa* species, addressing a significant data gap. We systematically identified the SOD gene family and revealed its evolutionary relationships through cross-species comparison. Further analysis of Dominin and CSRP homologs uncovered potential diversity in antioxidant defense mechanisms within this genus. Crucially, the next critical step involves rigorous functional validation of the identified SOD genes, for example, through gene expression analysis under oxidative stress, protein activity assays, or RNA interference experiments. These future functional assays, combined with genomic data from additional species, will not only validate our current findings but also further elucidate the evolutionary history of the SOD family in bivalves and the molecular basis of their environmental adaptation.

## Figures and Tables

**Table 1:** Summary of sequencing data quality.

Sample	Raw_Reads	Raw_Bases	Clean_Reads	Clean_Bases	Error_Rate	Q20	Q30	GC_pct
Hin1-S	22,938,901	6.88	22,567,525	6.77	0.01	98.10	94.44	39.17
Hin1-W	22,733,450	6.82	22,161,418	6.65	0.01	98.38	95.17	39.40
Hin1-B	22,769,642	6.83	22,389,164	6.72	0.01	98.37	95.06	40.64
Hin1-C	22,880,343	6.86	22,430,060	6.73	0.01	98.07	94.18	40.94
Hsp1-S	23,648,413	7.09	23,224,331	6.97	0.01	98.26	94.84	38.52
Hsp1-W	23,424,910	7.03	23,131,650	6.94	0.01	98.59	95.78	38.87
Hsp1-B	21,968,086	6.59	21,616,279	6.48	0.01	98.09	94.23	40.26
Hsp1-C	21,172,853	6.35	20,776,969	6.23	0.01	98.50	95.76	41.88
Hin2-S	23,079,592	6.92	22,625,841	6.79	0.01	98.17	94.59	38.83
Hin2-W	23,123,036	6.94	22,787,435	6.84	0.01	98.12	94.45	39.62
Hin2-B	23,654,582	7.10	23,320,078	7.00	0.01	98.28	94.74	40.92
Hin2-C	23,907,955	7.17	23,509,157	7.05	0.01	98.56	95.91	42.32
Hsi1-S	24,393,919	7.32	23,774,686	7.13	0.01	98.08	94.35	39.35
Hsi1-W	22,652,736	6.80	22,170,153	6.65	0.01	98.17	94.62	40.53
Hsi1-B	25,280,014	7.58	25,021,203	7.51	0.01	98.25	94.62	41.03
Hsi1-C	23,009,172	6.90	22,629,224	6.79	0.01	98.42	95.05	42.55
Hsi2-S	23,669,501	7.10	23,156,523	6.95	0.01	98.48	95.88	38.43
Hsi2-W	23,362,518	7.01	22,844,009	6.85	0.01	98.13	94.50	39.11
Hsi2-B	23,528,698	7.06	22,924,594	6.88	0.01	98.41	95.06	42.39
Hsi2-C	23,625,965	7.09	23,049,423	6.91	0.01	98.13	94.23	44.36

Note: S: gill, W: mantle, B: smooth muscle, C: striated muscle.

**Table 2:** Physicochemical properties of *Hyotissa* SOD proteins.

Sequence ID	Amino Acid	MW (kDa)	pI	Instability Index	Aliphatic Index	GRAVY	Cellular Location	Signal Peptide	Phosphorylation Sites
Hin1-DN145157c0g1i1.p1	161	16.57	5.94	25.20	77.45	−0.278	cyto	Other	11
Hin1-DN1483c1g1i7.p1	1035	111.91	6.41	35.72	80.09	−0.174	plas	Other	130
Hin1-DN12320c0g1i1.p1	351	37.04	6.70	32.90	63.56	−0.452	E.R.	SP (Sec/SPI)	21
Hin1-DN2804c1g1i4.p2	254	27.54	5.50	54.06	83.15	−0.393	extr	Other	21
Hin1-DN21488c0g1i1.p1	174	17.51	7.78	40.19	50.00	−0.563	cytonucl	Other	17
Hin1-DN636c0g1i2.p1	225	25.31	6.44	33.25	88.00	−0.288	mito	Other	21
Hsp1-DN3819c0g1i1.p1	161	16.62	5.94	25.13	75.03	−0.342	cyto	Other	11
Hsp1-DN109130c0g1i1.p1	154	15.94	5.70	21.62	78.44	−0.344	cyto	Other	7
Hsp1-DN9279c0g1i1.p1	1035	112.32	6.26	36.00	79.81	−0.204	plas	Other	129
Hsp1-DN4908c2g1i6.p1	247	26.84	5.66	50.45	82.75	−0.432	cyto	Other	21
Hsp1-DN4908c2g1i3.p1	254	27.56	5.36	53.34	82.01	−0.423	extr	Other	21
Hsp1-DN3853c0g1i1.p1	225	25.31	6.44	33.25	88.00	−0.288	mito	Other	21
Hin2-DN8896c0g1i4.p1	161	16.55	5.94	24.01	79.88	−0.232	cyto	Other	11
Hin2-DN27385c0g2i1.p1	154	15.94	5.70	21.62	78.44	−0.344	cyto	Other	7
Hin2-DN27385c0g1i1.p1	154	15.88	5.88	24.94	72.73	−0.421	cyto	Other	7
Hin2-DN252c1g4i1.p1	1035	111.97	6.28	37.21	80.10	−0.190	plas	SP (Sec/SPI)	129
Hin2-DN8896c0g1i3.p1	139	14.60	5.76	30.00	79.14	−0.156	cyto	Other	11
Hin2-DN20518c0g1i1.p1	351	36.96	6.70	34.66	62.19	−0.459	plas	SP (Sec/SPI)	21
Hin2-DN5802c0g1i2.p2	254	27.54	5.50	54.06	83.15	−0.393	extr	Other	21
Hin2-DN30196c0g1i3.p1	236	25.90	6.29	34.63	76.78	−0.371	cyto	Other	22
Hin2-DN19943c0g1i4.p1	174	17.51	7.78	40.19	50.00	−0.563	cyto	Other	17
Hin2-DN64c0g1i15.p1	225	25.31	6.44	33.25	88.00	−0.288	mito	Other	21
Hsi1-DN3539c0g2i1.p1	160	16.51	6.10	14.29	77.94	−0.293	cyto	Other	10
Hsi1-DN28797c0g1i1.p1	944	104.43	6.29	40.35	81.42	−0.293	plas	SP (Sec/SPI)	88
Hsi1-DN2474c0g1i7.p1	985	106.72	6.30	38.64	79.11	−0.234	plas	Other	124
Hsi1-DN2474c0g1i1.p1	1005	109.03	6.36	38.44	81.21	−0.195	plas	Other	126
Hsi1-DN2474c0g1i8.p1	1014	110.15	6.36	37.53	79.64	−0.209	plas	Other	128
Hsi1-DN28797c0g1i3.p1	907	100.56	6.05	40.16	78.83	−0.348	plas	SP (Sec/SPI)	86
Hsi1-DN2474c0g1i4.p1	682	73.65	6.09	37.49	84.68	−0.210	plas	Other	89
Hsi1-DN22897c0g1i1.p1	351	36.93	6.70	33.95	61.08	−0.465	plas	SP (Sec/SPI)	20
Hsi1-DN90c2g2i1.p1	292	32.67	6.79	44.89	65.75	−0.822	extr	SP (Sec/SPI)	27
Hsi1-DN28603c0g3i2.p1	296	31.16	8.75	29.60	52.40	−0.551	nucl	Other	30
Hsi1-DN28603c0g3i1.p1	525	54.59	9.75	41.41	47.39	−0.673	mito	SP (Sec/SPI)	24
Hsi1-DN15006c0g1i2.p1	197	21.26	5.15	46.29	91.32	−0.268	cyto	Other	13
Hsi1-DN129556c0g1i2.p1	192	21.14	5.10	42.66	72.71	−0.453	plas	SP (Sec/SPI)	12
Hsi1-DN2474c0g1i4.p2	298	32.57	6.74	41.25	66.38	−0.276	cyto	Other	34
Hsi1-DN3599c0g1i4.p1	225	25.29	6.63	36.78	88.44	−0.287	mito	Other	21
Hsi1-DN51085c0g1i2.p1	95	11.06	6.26	50.81	78.00	−0.612	cyto	Other	5
Hsi2-DN6176c0g1i1.p1	160	16.51	6.10	14.29	77.94	−0.293	cyto	Other	10
Hsi2-DN7547c0g1i12.p1	1032	112.09	6.42	35.77	82.68	−0.157	plas	Other	128
Hsi2-DN7547c0g1i8.p1	1012	109.79	6.36	35.91	80.66	−0.194	plas	Other	126
Hsi2-DN4644c0g2i4.p1	292	32.62	6.96	45.10	65.41	−0.809	extr	SP (Sec/SPI)	27
Hsi2-DN7796c0g1i1.p1	525	54.52	9.75	42.28	46.84	−0.677	mito	SP (Sec/SPI)	42
Hsi2-DN4644c0g2i2.p1	292	32.71	6.33	47.72	61.44	−0.847	extr	SP (Sec/SPI)	27
Hsi2-DN39370c0g1i1.p1	254	27.55	5.50	46.37	84.69	−0.404	extr	Other	20
Hsi2-DN2531c0g1i1.p1	225	25.29	6.63	36.78	88.44	−0.287	mito	Other	21

Note: cyto: Cytosol, plas: Plasma Membrane, E.R.: Endoplasmic Reticulum, cytonucl: Cytosol-Nucleus, mito: Mitochondria, nucl: Nucleus, extr: Extracellular.

## Data Availability

The raw transcriptome sequencing data have been deposited in the NCBI Sequence Read Archive (SRA) under accession number PRJNA1356962. The 16S rRNA sequence for species identification has been deposited in the NCBI database under accession numbers PX690962, PX690963, PX690964, PX690965, PX690966.

## Supplementary Materials

The following supporting information can be downloaded at https://www.mdpi.com/article/10.3390/biology15010004/s1, Table S1: Transcriptome statistics. Table S2: BUSCO completeness assessment. Table S3: Raw transcript expression levels (TPM) of SOD genes in *Hyotissa*. Table S4: Physicochemical properties of *Hyotissa* CSRP. Supplementary material provides the complete sequences (FASTA format) of all 46 superoxide dismutases, their predicted functional motifs and domain annotations, as well as the generated multiple sequence alignments and phylogenetic trees (Newick format).
