# Supplementary material for: Transcriptome Assembly and Comparative Analysis of the Superoxide Dismutase (SOD) Gene Family in Three Hyotissa Species
Source: Biology (Basel). 2025 Dec 19;15(1):4. doi: 10.3390/biology15010004 (PMC12784942; doi:10.3390/biology15010004)
Supplement: Supplementary file 1 [file biology-15-00004-s001.zip › Supplementary Table.docx]

**Table S1.** Transcriptome statistics.

| Sample | Number of transcripts | N50 (bp) |
| --- | --- | --- |
| Hin1 | 379502 | 1364 |
| Hsp1 | 333508 | 1595 |
| Hin2 | 358070 | 1405 |
| Hsi1 | 333550 | 1324 |
| Hsi2 | 327624 | 1439 |

Note:Hsi: *H. sinensis*, Hin: *H. inaequivalvis*, Hsp: *Hyotissa sp.*

**Table S2.**BUSCO Completeness Assessment.

| Sample | Total BUSCOs | Complete BUSCOs | Complete single-copy | Complete duplicated | Fragmented | Missing |
| --- | --- | --- | --- | --- | --- | --- |
| Hin1 | 5295 | 4756(89.8%) | 1948(36.8%) | 2808(53.0%) | 125(2.4%) | 414(7.8%) |
| Hsp1 | 5295 | 4940(93.3%) | 2035(38.4%) | 2905(54.9%) | 69(1.3%) | 286(5.4%) |
| Hin2 | 5295 | 4872(92.0%) | 1939(36.6%) | 2933(55.4%) | 90(1.7%) | 333(6.3%) |
| Hsi1 | 5295 | 4502(85.0%) | 1892(35.7%) | 2610(49.3%) | 194(3.7%) | 599(11.3%) |
| Hsi2 | 5295 | 4644(87.7%) | 2032(38.4%) | 2612(49.3%) | 130(2.5%) | 521(9.8%) |

Note:BUSCO completeness percentages across Mollusca lineages, including single-copy and duplicated genes.Hsi: *H. sinensis*, Hin: *H. inaequivalvis*, Hsp: *Hyotissa sp.*

**Table S3.** Raw transcript expression levels (TPM) of SOD genes in *Hyotissa*.

| Sequence ID | gill | mantle | smooth muscle | striated muscle |
| --- | --- | --- | --- | --- |
| Hin1-DN145157c0g1i1.p1 | 78.782602 | 84.321507 | 71.143911 | 140.370303 |
| Hin1-DN1483c1g1i7.p1 | 1.301498 | 1.583138 | 0.577772 | 0.862767 |
| Hin1-DN12320c0g1i1.p1 | 0 | 3.438416 | 0.443173 | 0.182605 |
| Hin1-DN2804c1g1i4.p2 | 1.576476 | 1.13434 | 3.425069 | 3.12273 |
| Hin1-DN21488c0g1i1.p1 | 0.226756 | 0 | 0.221366 | 0 |
| Hin1-DN636c0g1i2.p1 | 33.494382 | 42.717463 | 56.178498 | 161.442545 |
| Hsp1-DN3819c0g1i1.p1 | 61.689261 | 56.00278 | 143.399528 | 96.221693 |
| Hsp1-DN109130c0g1i1.p1 | 0 | 0 | 0 | 0.960281 |
| Hsp1-DN9279c0g1i1.p1 | 3.906181 | 5.100463 | 1.017105 | 1.752182 |
| Hsp1-DN4908c2g1i6.p1 | 0.603871 | 1.481886 | 0.190527 | 0.633227 |
| Hsp1-DN4908c2g1i3.p1 | 0.497737 | 0 | 0 | 0 |
| Hsp1-DN3853c0g1i1.p1 | 38.119256 | 32.516288 | 104.24901 | 121.731724 |
| Hin2-DN8896c0g1i4.p1 | 0 | 0 | 0.958702 | 0 |
| Hin2-DN27385c0g2i1.p1 | 0 | 0.728606 | 0.557898 | 0 |
| Hin2-DN27385c0g1i1.p1 | 0.079657 | 0.984698 | 0 | 0.178631 |
| Hin2-DN252c1g4i1.p1 | 4.461373 | 1.614321 | 2.107102 | 8.868372 |
| Hin2-DN8896c0g1i3.p1 | 0.851481 | 1.068214 | 1.975769 | 1.444089 |
| Hin2-DN20518c0g1i1.p1 | 0 | 0.076536 | 0 | 1.36125 |
| Hin2-DN5802c0g1i2.p2 | 2.93572 | 7.546693 | 4.551841 | 1.60581 |
| Hin2-DN30196c0g1i3.p1 | 0.071889 | 0.062033 | 0.067783 | 1.216691 |
| Hin2-DN19943c0g1i4.p1 | 0 | 0 | 0 | 0.877695 |
| Hin2-DN64c0g1i15.p1 | 0 | 0.858093 | 0.37714 | 0 |
| Hsi1-DN3539c0g2i1.p1 | 11.697829 | 6.826701 | 19.768596 | 25.150913 |
| Hsi1-DN28797c0g1i1.p1 | 0.039472 | 2.615887 | 0 | 0 |
| Hsi1-DN2474c0g1i7.p1 | 0 | 0.568782 | 0 | 0 |
| Hsi1-DN2474c0g1i1.p1 | 0.123452 | 1.675561 | 0 | 0 |
| Hsi1-DN2474c0g1i8.p1 | 0.629829 | 1.321792 | 0 | 0 |
| Hsi1-DN28797c0g1i3.p1 | 0 | 0.343548 | 0.036843 | 0.016548 |
| Hsi1-DN2474c0g1i4.p1 | 0.237467 | 0 | 0 | 0 |
| Hsi1-DN22897c0g1i1.p1 | 0.029418 | 0.873148 | 0 | 0 |
| Hsi1-DN90c2g2i1.p1 | 61.505868 | 2.489609 | 4.400175 | 23.800278 |
| Hsi1-DN28603c0g3i2.p1 | 0 | 0.60151 | 0 | 0 |
| Hsi1-DN28603c0g3i1.p1 | 0.060149 | 2.374526 | 0.092682 | 0.031417 |
| Hsi1-DN15006c0g1i2.p1 | 1.206474 | 0.892689 | 3.202331 | 4.363169 |
| Hsi1-DN129556c0g1i2.p1 | 0 | 0 | 0.377371 | 0 |
| Hsi1-DN2474c0g1i4.p2 | 0.237467 | 0 | 0 | 0 |
| Hsi1-DN3599c0g1i4.p1 | 7.11397 | 8.921997 | 1.980929 | 9.751633 |
| Hsi1-DN51085c0g1i2.p1 | 0 | 0 | 0 | 1.025895 |
| Hsi2-DN6176c0g1i1.p1 | 44.347538 | 28.718205 | 33.651916 | 17.392234 |
| Hsi2-DN7547c0g1i12.p1 | 0.511228 | 0 | 0.540581 | 0 |
| Hsi2-DN7547c0g1i8.p1 | 0.299954 | 0 | 0 | 0 |
| Hsi2-DN4644c0g2i4.p1 | 1.294752 | 0.405664 | 7.782852 | 0.281059 |
| Hsi2-DN7796c0g1i1.p1 | 1.356076 | 0 | 0.232439 | 0.163143 |
| Hsi2-DN4644c0g2i2.p1 | 0.180962 | 0 | 0.017121 | 0.076613 |
| Hsi2-DN39370c0g1i1.p1 | 1.327881 | 6.617273 | 1.950759 | 6.05864 |
| Hsi2-DN2531c0g1i1.p1 | 20.620171 | 47.149398 | 9.174298 | 35.536292 |

Note:Hsi: *H. sinensis*, Hin: *H. inaequivalvis*, Hsp: *Hyotissa sp.*

**Table S4.** Physicochemical properties of *Hyotissa* SOD proteins.

| CSRP Gene ID | Protein Length (aa) | Domain 1 (aa) | Domain 2 (aa) | Domain 3 (aa) | Domain4 (aa) |
| --- | --- | --- | --- | --- | --- |
| Hin1-DN1483c1g1i7.p1 | 1036 | 299-435 | 453-600 | 621-751 | 781-928 |
| Hsp1-DN9279c0g1i1.p1 | 1036 | 299-435 | 453-600 | 621-753 | 781-928 |
| Hin2-DN252c1g4i1.p1 | 1036 | 299-435 | 453-600 | 621-753 | 781-928 |
| Hsi1-DN28797c0g1i1.p1 | 945 | 323-461 | 475-623 | 644-794 | 802-940 |
| Hsi1-DN2474c0g1i7.p1 | 986 | 269-405 | 422-570 | 594-725 | 751-899 |
| Hsi1-DN2474c0g1i1.p1 | 1006 | 269-405 | 422-570 | 591-725 | 751-899 |
| Hsi1-DN2474c0g1i8.p1 | 1006 | 269-405 | 422-570 | 591-725 | 751-899 |
| Hsi1-DN28797c0g1i3.p1 | 908 | 323-461 | 475-623 | 644-794 |  |
| Hsi1-DN2474c0g1i4.p1 | 683 | 269-405 | 422-570 | 591-682 |  |
| Hsi2-DN7547c0g1i12.p1 | 1033 | 298-434 | 451-599 | 620-753 | 780-926 |
| Hsi2-DN7547c0g1i8.p1 | 1013 | 298-434 | 451-599 | 620-754 | 780-926 |

Note: All boundary coordinates were determined based on HMMER using the Pfam SOD domain model (PF00080) and refer to the amino acid (aa) positions within the respective protein sequences. Hsi1-DN28797c0g1i3.p1 and Hsi1-DN2474c0g1i4.p1 contain only three SOD domains; therefore, the fourth domain is left blank.
